# Editorial: Plant-microbe interactions and their role in salinity tolerance

**DOI:** 10.3389/fpls.2023.1142563

**Published:** 2023-01-30

**Authors:** Laura Pistelli, Marta Libik- Konieczny, Mirza Hasanuzzaman

**Affiliations:** ^1^ Department of Agriculture, Food and Environment, University of Pisa, Pisa, Italy; ^2^ The Franciszek Górski Institute of Plant Physiology, Polish Academy of Sciences, Kraków, Poland; ^3^ Department of Agronomy, Faculty of Agriculture, Sher-e-Bangla Agricultural University, Sher-e-Bangla Nagar, Dhaka, Bangladesh

**Keywords:** halophyte, endophytes, symbiotic microorganisms, bacterial community, stress elicitors, metabolome analysis, *Trichoderma*

Soil salinization is a major soil degradation process threatening the ecosystem and is recognized as one of the most important global problems for crop production, food security, and sustainable agriculture. Halophytes and halo-tolerant plants are adapted to natural saline environments, through physiological mechanisms such as regulation of cellular ion homeostasis and osmotic pressure, the detoxification of reactive oxygen species, and alterations in membrane composition and secondary metabolites production (Lombardi et al., 2022). To cope with salinity stress, much research has focused on producing stress-tolerant plant lines through genetic engineering and plant breeding. However, such methods have proven costly and time-consuming (Gupta et al., 2022).

One promising approach may be the introduction of salt-tolerant microbes to enhance plant growth in areas exposed to salinity stress. Both plant-associated fungi and bacteria hold great potential for improving plant nutrition, yield biomass, and stress tolerance. However, the contribution of symbiotic microbes to salt adaptation still needs to be understood entirely (Khalmuratova et al., 2020). Thus, combined data from genomic, transcriptomic, proteomic, and metabolomic studies would be essential to identify key pathways and processes controlled by microbial factors, resulting in plant tolerance to salinity. Studying the structure and function of microbial communities in saline soil could help elucidate their key role in the biological mechanisms of regulating nutrient cycles in saline soil (Zhang et al., 2019). It is thought that the co-evolution of halophytes and plant growth-promoting rhizobacteria (PGPR) has enabled these plants to survive in saline habitats (da Silva Folli-Pereira et al., 2013; Pan et al., 2020). Therefore, identifying microorganisms that thrive under different adverse environmental conditions can result in the discovery of beneficial symbiosis (Ma et al., 2020).

This Research Topic aimed to attract the attention of the scientific community to issues related to the influence of microorganisms on the physiological, biochemical and molecular mechanisms responsible for sensing, signaling, and responses to salt stress and drought stress in halophytes or halo-tolerant plants. Seven scientific papers have been published assessing the ability of plants to survive under saline conditions with symbiotic microorganisms.

The application of halotolerant inocula of *Glutamicibacter* sp. *or Pseudomonas* sp. during the growth of *Sueda fruticosa* under salt stress conditions significantly improved plant growth, positively influencing nutrient uptake and the activity of enzymatic antioxidants alleviating oxidative stress in the cells of this halophyte Hidri et al. In addition, inoculation with the bacteria influenced the indolyl-3-acetic acid (IAA) content of the rhizosphere and the activity of certain soil enzymes (e.g.urease and β-glucosidase), which play an important role in the geochemical carbon and nitrogen cycles and are responsible for nutrient availability. The increased levels of IAA regulated the lateral root initiation and root surface area, which helps plants to maintain sufficient nutrient uptake from the soil. Taken together it can be concluded that *Glutamicibacter* sp. and *Pseudomonas* sp. strains are promising candidates for use towards increased plant resistance to salt stress as well as phytoremediation of salinity-damaged soils.

In another research, Siddiqui et al. investigated the roles of plant– microbe interaction to improve the salt tolerance in *O. sativa* and *Z. mays*. They tested the salt tolerance potential of the endophyte collected from the root zone of halophytic grass. It was evident that endophyte-supplemented plants showed a substantial increase in growth, biomass, relative water content, antioxidant defense, and photochemical efficiency of plants. Their findings indicated that rice and maize could produce biomass for economic sustainability by using saline water irrigation on saline soils avoiding competition with conventional agriculture if supplemented with *A. terreus*. Therefore, exploring the potential endophytes and employing them as growth promotors and stress elicitors may be one of the efficient strategies for sustainable agriculture in the coastal ecosystem.

However, also the rhizobacteria isolated from the extreme environment in the Qinghai-Tibetan Plateau contributed to alleviating the stress of saline conditions in wheat cultivation (Wu et al., 2022). In this case, the plants inoculated with *Bacillus halotolerant* KKD1 showed increased seedling morphological parameters and physiological indexes. KKD1 was able to support plant growth under salt stress by inducing a stress response in wheat by modulating phytohormone levels, regulating lipid peroxidation, accumulating betaine, and excluding Na^+^. In addition, KKD1 positively affected the soil nitrogen content, soil phosphorus content and soil pH.

The resident leaf endophytic bacterial community of the olive tree was investigated to show their involvement in the salt stress adaptation of the plants, and the results are obtained by metabarcoding analyses (Vita et al., 2022). The authors assumed that mild stress level (100 mM NaCl) allowed the maintenance of an active transport of nutrients, water, and sodium inside the leaves, which may have favored the shift of endophytes towards specific bacteria taxa able to survive in this cellular context. Taxa as *Burkholderia* are more represented in this experimental condition could reinforce the hypothesis that the endophytic community may play a role in helping olive trees to deal with the harmful effects of sodium inside the leaves.

Limited research is committed to the role of fungal endophytes in saline conditions, although *Trichoderma harzianum* has already shown its beneficial effects on plants under normal and stressful conditions in addition to their role as biocontrol agents. Gupta et al. determined the metabolite and lipid changes in two fungal inoculated barley genotypes (the salt tolerant Vlamingh and the salt sensitive Gairdner). For both genotypes fungus-colonized plants exposed to NaCl (200 mM) had greater root and shoot biomass, and chlorophyll content than non-inoculated plants The researchers identified a total of 93 metabolites, of which 74 were identified in roots of both barley genotypes as organic acids, sugars, sugar acids, sugar alcohols, amino acids, amines, and a small number of fatty acids. They also detected 186 lipid molecular species of three major classes viz. glycerophospholipids, glycerolipids, and sphingolipids, from roots of both genotypes. These metabolites were also prominent in *Trichoderma*-inoculated plants and abundant in the Vlamingh tolerant genotype. Therefore, the stress-related metabolites induced by microbial inoculant plays a vital role in conferring salt tolerance (Gupta et al., 2022).

Many halophytes are used for the phytoremediation of environmental pollutants because of their physiological features. In their study, Supel et al. showed that the common ice plant, *Mesembryanthemum crystallinum*, which is an emi-halophyte and C_3_/CAM intermediate plant could tolerate high levels of soil Cd. When the plants were receiving some specific Cd concentrations, the CAM phase was induced by highly saline conditions. Eighteen bacterial and three yeast strains were isolated from the rhizosphere, which varied depending on the photosynthetic phases. After an *in vitro* test, five bacterial strains were selected and identified with a molecular proteomics technique; two strains of the species *Providencia rettgeri* were obtained from the C_3_ phase and three (one *Paenibacillus glucanolyticus* and two *Rhodococcus erythropolis* strains) from the CAM performing plants. Their results provided the notion of the synergic action of the plant together with the Cd/salt-resistant strains occurring within rhizospheral microbiota in ice plants.

The results of Wang et al. showed the ameliorating effect *of Sphingomonas* sh. Hbc-6 inoculation on maize plants growing under drought stress. The effect of inoculation was an increase in biomass and better performance in the tolerance to stress, as photosynthetic efficiency and chlorophyll content, a better osmoregulation due to osmolytes (proline and soluble sugar) and enhanced antioxidant machinery (enzymes and metabolites). Moreover, the maize rhizosphere community increased the richness and diversity, so that *Sphingomonas* sp. Hbc-6 strain can be used as a biofertilizer, especially in arid areas.

In our opinion, finding alternative strategies to genetic engineering and plant breeding techniques for plant growth, development and productivity under salt and drought stress conditions, as well as developing innovative technologies for managing and rehabilitating salt-affected environments, is an ongoing challenge for researchers.

## Author contributions

All authors listed have made a substantial, direct, and intellectual contribution to the work and approved it for publication.
